# Personalized Network Modeling of the Pan-Cancer Patient and Cell Line Interactome

**DOI:** 10.1200/CCI.19.00140

**Published:** 2020-05-06

**Authors:** Rupam Bhattacharyya, Min Jin Ha, Qingzhi Liu, Rehan Akbani, Han Liang, Veerabhadran Baladandayuthapani

**Affiliations:** ^1^ Department of Biostatistics, University of Michigan, Ann Arbor, MI; ^2^ Department of Biostatistics, The University of Texas MD Anderson Cancer Center, Houston, TX; ^3^ Department of Bioinformatics and Computational Biology, The University of Texas MD Anderson Cancer Center, Houston, TX; ^4^ Department of Systems Biology, The University of Texas MD Anderson Cancer Center, Houston, TX

## Abstract

**PURPOSE:**

Personalized network inference on diverse clinical and in vitro model systems across cancer types can be used to delineate specific regulatory mechanisms, uncover drug targets and pathways, and develop individualized predictive models in cancer.

**METHODS:**

We developed TransPRECISE (personalized cancer-specific integrated network estimation model), a multiscale Bayesian network modeling framework, to analyze the pan-cancer patient and cell line interactome to identify differential and conserved intrapathway activities, to globally assess cell lines as representative models for patients, and to develop drug sensitivity prediction models. We assessed pan-cancer pathway activities for a large cohort of patient samples (> 7,700) from the Cancer Proteome Atlas across ≥ 30 tumor types, a set of 640 cancer cell lines from the MD Anderson Cell Lines Project spanning 16 lineages, and ≥ 250 cell lines’ response to > 400 drugs.

**RESULTS:**

TransPRECISE captured differential and conserved proteomic network topologies and pathway circuitry between multiple patient and cell line lineages: ovarian and kidney cancers shared high levels of connectivity in the hormone receptor and receptor tyrosine kinase pathways, respectively, between the two model systems. Our tumor stratification approach found distinct clinical subtypes of the patients represented by different sets of cell lines: patients with head and neck tumors were classified into two different subtypes that are represented by head and neck and esophagus cell lines and had different prognostic patterns (456 *v* 654 days of median overall survival; *P* = .02). High predictive accuracy was observed for drug sensitivities in cell lines across multiple drugs (median area under the receiver operating characteristic curve > 0.8) using Bayesian additive regression tree models with TransPRECISE pathway scores.

**CONCLUSION:**

Our study provides a generalizable analytic framework to assess the translational potential of preclinical model systems and to guide pathway-based personalized medical decision making, integrating genomic and molecular data across model systems.

## INTRODUCTION

Precision medicine aims to improve clinical outcomes by optimizing treatment to each individual patient. The rapid accumulation of large-scale panomic molecular data across multiple cancers on patients (the International Cancer Genome Consortium,^1^ the Cancer Genome Atlas [TCGA],^2^ Pan-Cancer Analysis of Whole Genomes [PCAWG],^3^ the Cancer Proteome Atlas [TCPA]^4,5^) and model systems (Genomics of Drug Sensitivity in Cancer [GDSC],^6^ Cancer Cell Line Encyclopedia [CCLE],^7^ MD Anderson Cell Lines Project [MCLP]^8^), together with extensive drug profiling data (NCI60 [National Cancer Institute-60 Human Tumor Cell Lines Screen],^9^ the National Institutes of Health Library of Integrated Network-Based Cellular Signatures,^10^ Connectivity Map,^11-13^ The Cancer Dependency Map Project^14^) have generated information-rich and diverse community resources with major implications for translational research in oncology.^15^ However, a major challenge remains: to bridge anticancer pharmacologic data to large-scale omics in the paradigm wherein patient heterogeneity is leveraged and inferred through rigorous and integrative data-analytic approaches across patients and model systems.

**CONTEXT**

**Key Objective**
Integrative analyses of molecular data across patient tumors and model systems offer insights into the translational potential of preclinical model systems and the development of personalized therapeutic regimens.
**Knowledge Generated**
We present TransPRECISE (personalized cancer-specific integrated network estimation model), a network-based tool to assess pathway similarities between patients and cell lines at a sample-specific level. Using proteomic data across multiple tumor types, TransPRECISE identified several key pathways linking patient tumors and cell lines (eg, receptor tyrosine kinase in kidney cancers, hormone signaling in ovarian cancers, and epithelial–mesenchymal transition pathway in melanoma and uterine cancers). Using predictive models trained on cell lines, TransPRECISE predicted high response rates for several known drug-cancer combinations (eg, ibrutinib in patients with breast cancer and lapatinib in patients with colon cancer).
**Relevance**
The TransPRECISE framework has potential use in identifying appropriate preclinical models for prioritizing specific drug targets across tumor types and in guiding individualized clinical decision making.

Complex diseases such as cancer are often characterized by small effects in multiple genes and proteins that are interacting with each other by perturbing downstream cellular signaling pathways.^16-18^ It is well established that complex molecular networks and systems are formed by a large number of interactions of genes and their products operating in response to different cellular conditions and cell environments (ie, model systems).^19^ To date, most, if not all, approaches to mechanism and drug discovery have been constrained by the biologic system^20,21^ (patients or cell lines), specific cancer lineage,^22,23^ or prior knowledge of specific genomic alterations.^24,25^ Hence, there is a critical need for robust analytic methods that integrate molecular profiles across large cohorts of patients and model systems from multiple tumor lineages in a data-driven manner to delineate specific regulatory mechanisms, uncover drug targets and pathways, and develop individualized predictive models in cancer.

We have recently developed a network-based framework called PRECISE (personalized cancer-specific integrated network estimation model) to estimate cancer-specific networks, infer patient-specific networks, and elicit interpretable pathway-level signatures.^26^ Using a large cohort of patients (> 7,700) from TCGA across ≥ 30 tumor types, we have shown that PRECISE identifies pan-cancer commonalities and differences in proteomic network biology within and across tumors, allows robust tumor stratification that is both biologically and clinically informative, and has superior prognostic power compared with multiple existing approaches.^26^ In this article, we present translational PRECISE (TransPRECISE, in short), a generalization of the PRECISE framework, to establish the translational relevance of these pathway signatures. Briefly, TransPRECISE uses a multiscale Bayesian modeling strategy that infers de novo differential and conserved networks of intrapathway circuitry between the two biologic systems (patients and cell lines) for multiple cancers. Furthermore, it identifies cell-line “avatars” for patients based on pathway activities and develops machine learning–based predictive models for drug sensitivity in both cell lines and patients to potentially guide pathway-based individualized medical decision making. We have also developed an online, publicly available, comprehensive, interactive database and visualization tool of our findings, together with software code.^27^

## METHODS

### Proteomic Data on Patients With Cancer

We used a data set of 7,714 patient samples across 31 different cancer types available from TCPA;^4,5^ (Data Supplement). TCPA offers reverse-phase protein array (RPPA)–based proteomics data sets, profiled using extensively validated antibodies to nearly 200 proteins and phosphoproteins. The functional space of the antibodies covers major functional and signaling pathways relevant to human cancers. For this work, we used a total of 12 pathways, including DNA damage response, epithelial–mesenchymal transition (EMT), hormone signaling, apoptosis, tuberous sclerosis complex/mammalian target of rapamycin (TSC/mTOR), and RAS/mitogen-activated protein kinase (MAPK; Data Supplement).

### Cancer Cell Lines’ Proteomic and Drug Sensitivity Data

We used RPPA-based protein expression data for cell lines available via the MCLP.^8^ In a set of 640 cancer cell lines spanning 16 lineages, each cell line has RPPA expression data that are based on the same set of proteins as in the patient tumors (Data Supplement). In addition, we used drug sensitivity data from the GDSC^6^ database, with the sensitivity of 481 drugs assessed on a subset of 254 cell lines (Data Supplement). In this article, we will denote cell line samples in lowercase and patient samples in uppercase letters.

### TransPRECISE Framework

**FIG 1.**
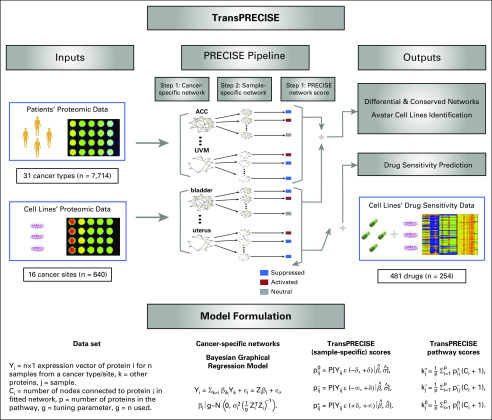
Overview of the TransPRECISE framework. The first step of TransPRECISE involves implementing the PRECISE pipeline on two sets of RPPA protein expression data –namely, cancer patients (7,714 samples across 31 different cancer types) and cancer cell lines (640 samples across 16 different cancer tissues). For each combination of 47 cancer types across cell lines and patients and the 12 pathways, the PRECISE procedure is executed in three consecutive steps: fitting cancer-specific protein networks using Bayesian graphical regression (step 1); deconvolving these cancer-specific networks to fit sample-specific pathways networks (step 2); and aggregating the sample-specific networks to obtain calibrated TransPRECISE scores and pathway activity status (step 3). The cancer-specific networks from step 1 are compared across patients and cell lines for each pathway for pan-cancer identification of differential and conserved pathway activities. The TransPRECISE scores from steps 2 and 3 are used to identify potential avatar cell lines and the lineages for patient tumors and to construct prediction models for drug sensitivity trained in in vivo drug sensitivity and used for in silico drug sensitivity prediction of patients′ drug response. The bottom panel provides the details and equations for the computational steps of the Bayesian graphical regression procedure and post-processing of the regression outputs to obtain the cancer-specific and sample-specific summaries. All probabilities are computed under the fitted Bayesian graphical regression model with the estimated parameters, with the superscripts 0, +, and −, respectively corresponding to the neutral, activated, or suppressed status of the pathway. The *p_ij_*s are the posterior probabilities corresponding to protein i and sample j, and the *k_j_*s are the aggregated pathway scores for sample j.

The TransPRECISE implementation can be classified broadly into 3 modules (Fig 1). The first module takes as input the combined proteomics data from patients and cell lines (as described earlier in the text). The second module implements the PRECISE modeling framework, providing the cancer-specific pathway networks and sample-specific pathway scores as outputs. The final module predicts patient drug responses on the basis of models trained on the cell lines. The model-specific parameterization and inferential strategies are described in the Data Supplement.

## RESULTS

### Differential and Conserved Rewiring and Circuitry of Cancer-Specific Networks

Using the de novo cancer-specific population-level networks (from step 1 of TransPRECISE), we evaluated intrapathway edge rewiring (Data Supplement) across lineages of the two model systems to identify highly conserved and differential edges and to link patient and cell line tumor types by measuring intrapathway circuitry.

#### Network rewiring across model systems.

**FIG 2.**
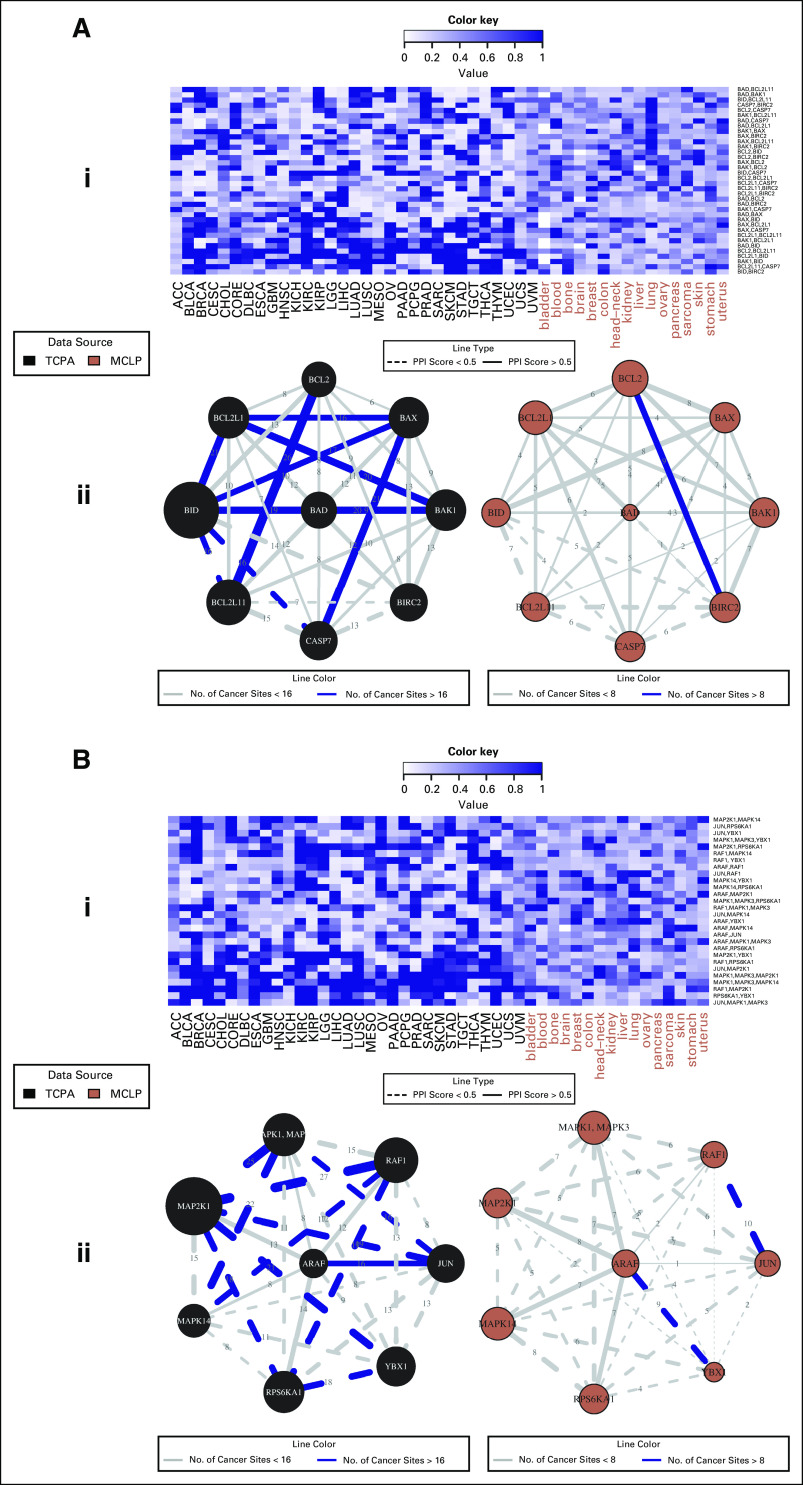
Pan-cancer summary of protein networks for apoptosis (A) and RAS/MAPK (B) pathways. i. Heatmap depicting strengths of all possible protein-protein edges within the pathway, across all 47 patient and cell line tumor lineages, quantified by the posterior inclusion probabilities of the edges based on the fitted Bayesian graphical regression model. ii. Networks depicting pan-cancer commonalities and differences in cancer-specific network structures: edges are weighted by the edge consistencies, which are quantified by the number of patient tumor types holding that particular edge with a posterior probability (PPI) >0.5, and labeled by solid lines if the edges are confirmed by the interaction scores from STRING database. The left and right panels are networks for patients and cell lines, respectively. MCLP, MD Anderson Cell Lines Project; TCPA, the Cancer Proteome Atlas.

We determined the extent to which protein-protein edges in each of the pathways were shared across tumor sites in the patients and the cell lines. We found highly conserved edges across lineages for both cell lines and patients (Fig 2 and Data Supplement). All of the 12 pathways had at least one link that was shared across more than 20 lineages among the patient cancer types, and 11 pathways (with the exception of hormone signaling) had at least one link that was shared across more than eight lineages among the cell line lineages. The conserved edges were further classified into three categories: (1) patient cell lines, (2) patients only, and (3) cell lines only. For category 1, we identified a significant correlation of CCNE2-FOXM1 (10 cell line lineages, 17 patient cancer types) in cell cycle CTNNB1-SERPINE1 (eight cell line lineages, 17 patient cancer types) in EMT, and RB1-RPS6 (eight cell line lineages, 20 patient cancer types) in TSC/mTOR pathways.

#### Linking tumor types between model systems on the basis of network circuitry.

**FIG 3.**
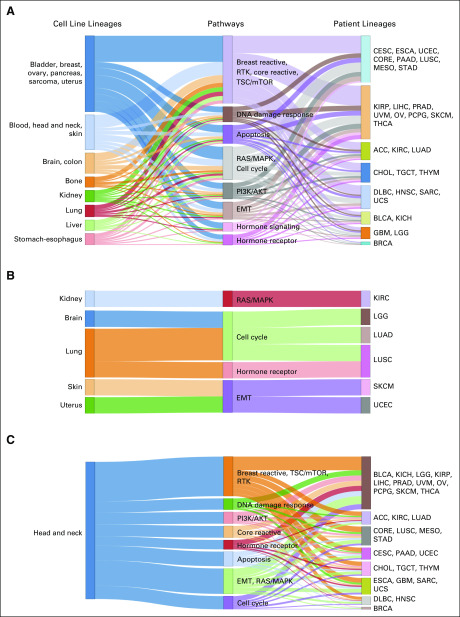
Sankey diagrams for patient and cell line cancers with conserved pathway-specific connectivity. (A) The columns contain cell line cancers, pathways, and patient cancers from left to right, respectively. A cell line cancer tissue is connected to a pathway if the connectivity score (CS) for that cancer type-pathway pair (defined as the proportion of edges out of all possible undirected edges in the pathway that are held by that cancer type) is more than 900 out of 1,000 random CS values computed for that cancer type, with repeated random selection of the same number of proteins as in the pathway from the pool of all proteins across the 12 pathways. The connection between a patient cancer type to a pathway is also determined by the same rule. The length of the middle (pathway) column pieces indicate the participation of that pathway in driving the conservation across the two model systems. As seen in panel A, ovary and uterus cell lines were connected via the hormone signaling (breast) pathway with *BRCA*; lung, kidney, and stomach-esophagus cell lines were linked together with two clusters of patient cancers (*KICH, KIRP, PRAD, LGG and LUSC, UCEC, STAD*) via the RTK pathway. (B) The Sankey diagram contains only the subset of cell line cancer (ie, patient cancer pairs that have same tissue-specific lineage), and the cutoff for CS values is higher than 800 of the 1,000 random CSs obtained using the random selection of proteins. Panel B presents clear confirmations of conservation of activities across model systems within cancer tissues, some specific examples being bladder-core reactive (BLCA), kidney (RTK-KICH and KIRP), kidney-hormone receptor (KIRC), ovary-hormone signaling (OV), and stomach-hormone receptor (ESCA and STAD). (C) The Sankey diagram contains only the subset of the edges that are originating from the head and neck cancer cell line type, and the cutoff for CS values is higher than 800 of the 1,000 random CSs obtained using the random selection of proteins.

We investigated the shared cross-signaling between cell line and patient tumor types. As a measure of the level of cross-signaling (Data Supplement) of a specific pathway network, we defined the connectivity score (CS) as the ratio of the observed number of edges in a given network to the total number of possible edges in the pathway, because more edges imply a higher level of cross-signaling within a pathway (Data Supplement). In addition, we quantified the level of significance for the observed CS value by comparing it with CS values obtained from random permutation of the network, called randomCS; lower values of randomCS provide evidence against the observed CS value being obtained under random chance (Data Supplement). On the basis of the randomCS, we evaluated the similarity between cell line and patient tumor types in terms of network cross-signaling. Specifically, we declared two lineages were similar for a pathway if both of them showed high levels of cross-signaling (ie, low randomCS proportions). Some key triplets of cell line/pathways/patient are summarized in Figure 3.

### Pan-Cancer Stratification Across Model Systems on the Basis of TransPRECISE Scores

We deconvolved the global population-level networks to obtain sample-specific pathway-level functional summaries of the proteomic crosstalk within a pathway; in other words, for a given pathway, each sample has three different scores for activated, neutral, and suppressed statuses of the pathway. For tumor stratification, we used the network aberration score, defined as the sum of the activated and suppressed TransPRECISE scores for each sample.

**FIG 4.**
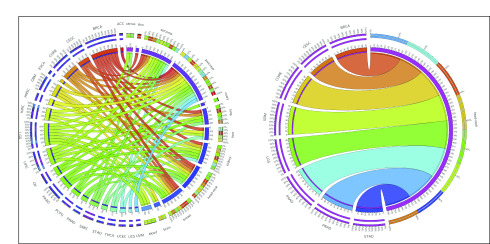
Circos plots summarizing high correlations of network aberration scores between patient and cell line cancers. (A) An edge exists between a patient cancer type and a cell line cancer lineage if more than 75% of all possible patient-cell line pairs for that pair of cancers have a Pearson correlation of magnitude 0.9 or higher between their sets of the 12 pathway network aberration scores (sum of TransPRECISE sample-specific pathway activation and suppression scores). The edge strengths are determined by these percentages, as well. The edge colors indicate the patient cancers from which the edge originates, and the lengths of the innermost node pieces indicate the neighborhood size of the corresponding node. The two circular axes in the exterior indicate relative strengths of the edges originating from the same node, and the sections are colored by the opposite node to which that edge is connected, with the edges now arranged according to decreasing order of strength. (B) This panel contains the subset of the plot in Panel A with only the connections originating from the head and neck cell line type visible.

For linking cell lines and patients, we computed the Pearson’s correlation for aberration score vectors (across 12 pathways) from each cell line–patient pair. The majority of the cell line–patient pairs for sarcoma-SARC (green), kidney-KIRC (light green), breast-BRCA (orange), and brain-LGG and -GBM (light green and yellow; edge colors in Figure 4 parenthesized) showed absolute correlations > 0.9. Interestingly, pancreatic and brain cancers were highly correlated across model systems: 99% of pancreas-HNSC pairs and 93% of GBM-pancreas pairs (and also 92% of the PAAD–head and neck pairs) had absolute correlations > 0.9, and most of these connections seem to be driven by high aberration scores in the DNA damage response pathway (Data Supplement).

**FIG 5.**
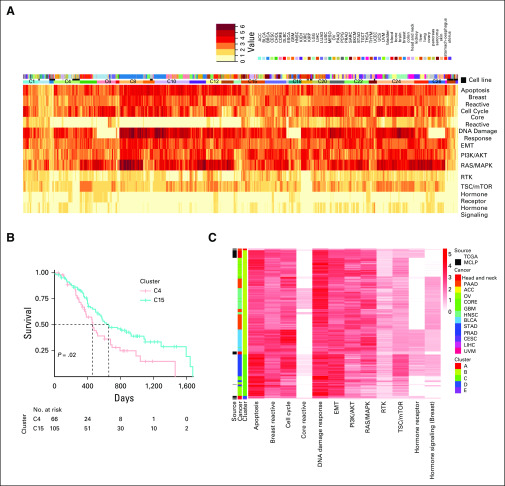
Avatar cell lines identification and selection of driving pathways using network aberration scores. (A) Heatmap depicting network aberration scores (combined activation and suppression TransPRECISE pathway scores) after running unsupervised hierarchical clustering of the score matrix consisting of 8,354 samples (7,714 patients across 31 cancer lineages and 640 cell lines across 16 cancer types) and 12 proteomic signaling pathways. Twenty-nine clusters are identified by gap statistic. Out of the three annotation bars, the topmost one indicates tumor types, the middle one indicates whether the sample is a patient or a cell line, and the bottom one indicates cluster participation according to which the samples are grouped. (B) Kaplan-Meier curves depicting difference between survival times of patients with head and neck squamous cell cancer grouped in clusters C4 and C15 using the hierarchical clustering method on TransPRECISE network aberration scores. (C) Heatmap depicting network aberration scores (combined activation and suppression TransPRECISE pathway scores) after running unsupervised hierarchical clustering of the score matrix consisting of all patient samples and only the head and neck cell line samples across the 12 pathways. Out of the three annotation bars, the leftmost one indicates whether the sample is a patient or a cell line, the middle one indicates the cancer type, and the rightmost one indicates cluster participation according to which the samples are grouped.

To find robust pan-cancer stratification across model systems, we applied hierarchic clustering using the complete linkage method^28^ on the correlations of the aberration scores. Among the 29 optimal clusters across patients and cell lines (Fig 5), most of the cell lines have a mixed membership; eight clusters (C2, C3, C4, C9, ^13^C, C14, C19, and C23) have patient tumors, whereas cluster C29 includes only cell lines (48 out of 640 in total, 7.5%). Cluster C4 showed a high level of fidelity in lineages between cell-line and patient tumor types; it includes 81% of ovary cell lines and 11% of patients with ovarian cancer (OV), 72% of head and neck cell lines and 38% of patients with HNSC, and 20% of pancreas cell lines (another 70% of them being located in C2 with notable aberration of the RAS/MAPK pathway) and 80% of patients with PAAD, exhibiting high aberration in apoptosis and DNA damage response pathways (Data Supplement). Within cluster C4, we observed significant correlations between the patient–cell line samples from ovary-PAAD, OV, BLCA, skin-PAAD, and head and neck–BLCA, HNSC (Data Supplement). More specifically, the HNSC samples were almost exclusively divided into the 2 clusters, C4 (n = 78 [38%]) and C15 (n = 122 [60%]), that include 38 head and neck cell lines (73%) and five esophagus cell lines (100%), respectively (Data Supplement). The co-occurrence of squamous cell carcinoma of the head and neck and esophageal cancer is not uncommon.^29,30^

### Characterization of Head and Neck Cancer Cell Lines and Patients

We focused on a case study using only the head and neck cell lines in conjunction with all the patient samples from TCGA. As presented in Figure 3C, we observed connections from the head and neck cell lines to the patient cancers across the pathways at a threshold of randomCS proportion < 0.2. One significant observation is that the head and neck cell lines are connected to the HNSC samples via several pathways including receptor tyrosine kinase (RTK), apoptosis, cell cycle, and EMT. Notably, the set of patient cancers for which at least 75% of the sample-sample pairs with the head and neck cell lines have highly correlated network aberration scores across all pathways includes the BRCA, CORE, LGG, and GBM samples but does not include the HNSC samples, which is in line with the findings presented in Figure 3C because those connections were stronger than the connection with HNSC (Fig 4B). In hierarchic clustering of the head and neck cell lines and all the patient samples, a subset of the head and neck cell lines cluster with a subset of the patients with HNSC with high aberration in the DNA damage response pathway. In the hierarchic clustering on the basis of all patients and cell lines, we found a significant difference in survival outcome between patients with HNSC in C4 and those with HNSC in C15: the median survival was 456 days and 654 days for C4 and C15, respectively, with a *P* value of .02 (Fig 5B). The patients in C15 who were represented by esophagus cell lines showed better survival than did those in C4, which includes head and neck cell lines; this indicates that our TransPRECISE scores captured distinct prognostic information in patients with HNSC. Moreover, the patterns of pathway activity and status were significantly different between the two clusters. The patients with HNSC in both C4 and C15 had high aberration scores in apoptosis, PI3K/AKT, and DNA damage response pathways. Specifically, for the DNA damage response pathway, the two clusters exhibited significantly distinct TransPRECISE statuses; 72% of patients in C4 showed suppression and 65% of patients in C15 showed activation (χ^2^ test *P* < .0001).

### Drug Response Prediction Using TransPRECISE Scores

#### Training drug response prediction models in cell lines.

For the subset of cell lines in which drug sensitivity data are available (Data Supplement), we used Bayesian additive regression trees (BART),^31^ a machine learning method, to build predictive models from the network aberration scores for the 12 pathways. For each cancer, we fit BART, with drug response (sensitive or resistant), as a binary outcome and TransPRECISE scores as predictors, for the drugs having profiles of ≥ 10 cell lines for that cancer type.

**FIG 6.**
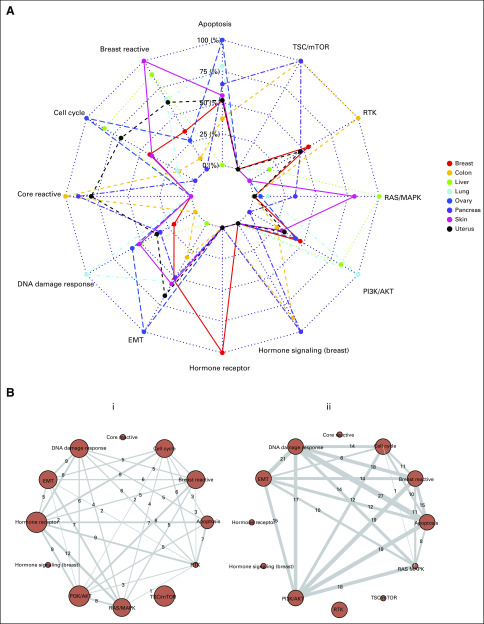
Performance of pathways in drug response prediction for cell lines across cancer lineages, based on test-set area under the curve (AUC) values evaluated from five-fold cross validation. (A) For a tissue type, we only look at the subset of drugs for which we have at least 10 response profiles from cell lines in that lineage and at least 0.85 test-set AUC using a five-fold cross-validation in the BART models. Then, for each pathway, we compute the proportion of times it is the top predictor in models for such drugs. The radar plot shows these proportions in a log_e_ (1+.)-transformed scale. The significance and ranking of each of the twelve pathways in a model are quantified by posterior probabilities of inclusion in such a final predictive model for drugs. (B) Networks showing the number of times (within models satisfying the criteria in panel A) a pair of pathways are the top two predictive pathways in a BART model. Panel i is for the breast cancer cell lines, and panel ii is for the lung cancer cell lines.

We found that TransPRECISE scores conferred high predictive power, translating to high median test-set areas under the receiver operating characteristic curves (AUCs) across the lineages; all lineages had median AUCs > 0.8, with lung, breast, and colon being the top 3, having median AUCs > 0.9 (Data Supplement). From the radar plot summarizing the top pathway predictors across all drugs for each lineage (Fig 6A), we observed some notable evidence of predictive affinity for certain pathways to specific lineages: hormone receptor in breast; core reactive, RTK, and TSC/mTOR in colon; RAS/MAPK in liver; DNA damage response and PI3K/AKT in lung; apoptosis, cell cycle, and EMT in ovary; and DNA damage response and TSC/mTOR in pancreas cell lines. Furthermore, we investigated pathway interaction in predicting drug sensitivity (Fig 6B). The breast cancer–related pathways and breast reactive and hormone receptor pathways were highly synergistic in predicting the responses of five drugs including ML311 in breast cancer cell lines.^32^

#### Predicting drug sensitivity in patient tumors.

For each cell-line cancer lineage, for which the training models were fitted with the TransPRECISE pathway scores (as described earlier in the text), we predicted drug sensitivity in patient tumors within matched tissue types (a total of 10 lineages). We found drugs that had 100% response rates, especially in BRCA, CORE, LIHC, PAAD, and SKCM, some of which are under clinical investigations in their respective cancers (Data Supplement). For example, all patients with BRCA were predicted to be responsive to ibrutinib, which targets Bruton tyrosine kinase with RAS/MAPK, PI3K/AKT, and EMT as the top predictive pathways (Data Supplement). Using patient drug exposure data from the Gene-Drug Interactions for Survival in Cancer (GDISC) database, we evaluated the models’ predictive performances (Data Supplement).^33,34^ For all the CORE patients, our model, trained on the colon cell lines for the drug lapatinib, predicts the true exposure correctly (note the same drug-cancer combination was also predicted to have a 100% response). Furthermore, for > 90% of the patients with OV, our model fitted on the ovary cell lines managed to correctly predict the response to the drug paclitaxel, which, by current standards, remains an integral part of the chemotherapeutic treatment of OV.^35-37^

## DISCUSSION

The investigation of patient tumors and cell line interactome offers insights into the translational potential of preclinical model systems. This requires the development of analytic models that capture the molecular heterogeneity of a cancer type in an unbiased manner and accurate calibration of aberrant biologic pathways. We propose TransPRECISE, a multiscale Bayesian network modeling framework, whose overarching goals are 3-fold: to identify differential and conserved intrapathway activities between two different model systems (patient tumors and cell lines) across multiple cancers; to globally assess cell lines as representative in vitro models for patients on the basis of their inferred pathway circuitry; and to build drug sensitivity prediction models for both cell lines and patients to aid pathway-based personalized medical decision making. To the best of our knowledge, TransPRECISE is the first computational approach that provides a conflation of these goals.

In this proof-of-concept study, we illustrate the utility of TransPRECISE using RPPA-based proteomic expression profiles from patients and cell lines across several functional pathways, and the cell lines’ drug response. The protein interactions that were present in both model systems offer valuable insights into the shared pathway circuitry across model systems, which has potential translational usefulness in studying the role of the tumor microenvironment. For example, the robust link CCNE2-FOXM1 within the cell cycle pathway has been identified as having important implications in the modulation of several cancers, such as breast,^38^ prostate cancer subtype 1,^39^ hepatocellular carcinoma,^40^ and osteosarcoma.^41^ The aberration of the highly shared edge CTNNB1-SERPINE1 in the EMT pathway has been found to affect the growth of malignant cell masses in several cancers, including cancers of the gastric system,^42,43^ pancreatic cancer,^44^ and breast cancer.^45^ We also found a high degree of fidelity to their histologic sites between model systems based on the level of network cross-signaling (eg, the RTK pathway in kidney cancers^46,47^ and the hormone signaling pathway in OV).^48,49^ As additional validation, TransPRECISE implicated cross-signaling in the EMT pathway in SKCM and UCEC, which is expected because the SKCM cohort contains many metastatic samples^50^ and UCEC includes epithelial-like endometrioid samples as well as mesenchymal-like serous samples.^51^ TransPRECISE implicated the hormone receptor pathway in lung cancer, which is another known observation that is being studied for its translational potential.^52^ Our sample-specific inference of pathway activity provided robust tumor stratification across model systems that include distinct prognostic information (Fig 5). These robust edges and cross-signaling of pathways across model systems and cancer sites will potentially provide complementary information in terms of disease characterization and therapeutic targets.

Our Bayesian prediction models using the pathway scores on a cell line’s drug sensitivity provided high prediction accuracies (median test-set AUC > 0.8 across all drugs and all cancers) and selected cancer-specific pathway signatures in predicting drug response, such as hormone receptor–breast,^53^ and TSC/mTOR–pancreas.^54,55^ Our training models using cell lines were used to predict patients’ drug response and validated with their known sensitivities. For example, ibrutinib, which had high predicted sensitivity for all the BRCA samples, has been investigated for its impact on human epidermal growth factor receptor 2 (HER2)–amplified breast cancers.^56^ Similarly, lapatinib, in combination with trastuzumab, has recently been tested clinically for HER2-amplified metastatic colorectal cancer.^57^

The TransPRECISE algorithm can be generalized to any disease system that provides matched genomic or molecular data on model and primary patient samples. For example, the transition from RPPA to other advanced high-throughput platforms and the development of databases, such as CPTAC,^58^ open up the opportunity to include more proteins (thus, more pathways) in the network analyses, leading to a more global coverage of the proteomic crosstalk between model systems. Furthermore, the PRECISE^26^ pipeline, which lies at the core of TransPRECISE analyses, allows the integration of upstream regulatory information and multiomics layers such as mutations, copy number, methylation, and mRNA expression. These modalities can be leveraged for better and holistic rewiring of pathway circuitry. Finally, our framework can be applied, in principle, to emerging model systems, such as patient-derived xenografts^59,60^ and organoids,^61^ that allow better recapitulation of the human tumor microenvironment. In summary, TransPRECISE offers the potential to bridge the gap between human and preclinical models to delineate actionable cancer-pathway-drug interactions to assist personalized systems biomedicine approaches in the clinic.

## DATA AVAILABILITY

We have created an online, publicly available R shiny app (available at https://bayesrx.shinyapps.io/TransPRECISE/) that is a comprehensive database and visualization repository of our findings. All codes used in generating our results are available, along with the documentation, on https://github.com/bayesrx/TransPRECISE.

## 
